# Sequence Permutation Generated Lysine and Tryptophan-Rich Antimicrobial Peptides with Enhanced Therapeutic Index

**DOI:** 10.3390/antibiotics14111077

**Published:** 2025-10-26

**Authors:** Kuang-Li Peng, Yu-Hsuan Wu, Hsuan-Che Hsu, Jya-Wei Cheng

**Affiliations:** Department of Medical Science, Institute of Biotechnology, National Tsing Hua University, Hsinchu 300, Taiwan; s107080901@m107.nthu.edu.tw (K.-L.P.); s112080801@m112.nthu.edu.tw (Y.-H.W.); s112080591@m112.nthu.edu.tw (H.-C.H.)

**Keywords:** antimicrobial peptide, drug design, sequence permutation, lysine, tryptophan, therapeutic index, wound healing

## Abstract

**Background/Objectives:** Antimicrobial peptides (AMPs) are promising therapeutic agents due to their broad-spectrum activity against bacteria, viruses, and fungi. Unlike traditional antibiotics, AMPs target microbial membranes directly and are less likely to induce resistance. They also possess immunomodulatory and wound-healing properties. However, clinical application remains limited by factors such as salt sensitivity, low bioavailability, and poor stability. To address these challenges, researchers have turned to structural optimization strategies. Recently, artificial intelligence (AI) has facilitated peptide drug design by rapidly screening large peptide libraries. Still, AI struggles to predict how subtle sequence changes affect peptide structure and function. Traditional sequence permutation offers a complementary approach by analyzing structural and functional effects without altering amino acid composition. **Methods:** In this study, we applied a clockwise sequence permutation strategy to the AMP W5K/A9W, generating derivative peptides with identical molecular weight, net charge, and hydrophobicity. We aimed to investigate how lysine and tryptophan distribution affects antimicrobial activity, membrane permeability, and selectivity. We assessed the secondary structures using circular dichroism (CD) spectroscopy and evaluated *in vitro* antimicrobial activity, salt resistance, membrane-permeabilizing ability, hemolysis, and wound healing effects. **Results:** The results revealed that the sequence arrangement of key residues significantly impacts peptide bioactivity and therapeutic index. **Conclusions:** This study highlights the importance of sequence order in determining AMP function. It also supports integrating permutation strategies with AI-based design to enhance AMP discovery. Together, these approaches offer new opportunities to combat drug-resistant pathogens and advance next-generation anti-infective therapies.

## 1. Introduction

Antimicrobial peptides (AMPs) are a promising class of antimicrobial agents, attracting growing attention in recent years due to their broad-spectrum activity against a wide range of pathogens, including bacteria, viruses, and fungi [1,2,3]. These peptides are produced by various organisms, from bacteria to mammals, and represent a critical component of the innate immune system’s first line of defense against invading pathogens [4,5,6]. Compared to traditional antibiotics, AMPs offer several advantages, such as targeting microbial membranes and a lower likelihood of inducing resistance [7,8]. Moreover, many AMPs exhibit immunomodulatory and wound healing properties, making them attractive candidates for the development of novel infectious disease therapies [9,10].

Despite their potential, the therapeutic application of AMPs faces several challenges, including salt sensitivity, high synthesis cost, low bioavailability, and poor stability [3,11,12,13]. Salt sensitivity is closely related to the bactericidal mechanism of AMPs, and it has been observed that clinically active peptides such as human β-defensin-1 and P-113 lose significant activity under high-salt conditions [14,15]. Similar issues have been reported for other AMPs as well [16,17]. Therefore, extensive research has focused on improving the effectiveness of salt-sensitive AMPs through chemical modification strategies [18,19,20].

Currently, artificial intelligence (AI) has been widely applied in drug discovery, particularly in peptide design and screening processes [21,22,23,24,25,26]. Deep learning models such as TransSAFP have dramatically accelerated the pace of development by enabling high-throughput predictions of activity and structural potential for thousands of amino acid sequences [24]. However, despite AI’s powerful computational capabilities, traditional design approaches—especially sequence permutation and functional validation—still play an irreplaceable role [25,27]. Sequence permutation design emphasizes evaluating how rearranging the order of amino acids, while keeping the composition constant, influences biological activity [28,29]. It is a rational and experimentally verifiable design strategy. Although AI can assist in sequence selection, it often struggles to accurately simulate or predict the functional consequences of subtle sequence variations [25]. In contrast, conventional methods allow for systematic verification of each permutation’s biological properties, thereby constructing a reliable sequence-function relationship—something AI alone cannot fully achieve.

Wang et al. used the tryptophan-rich peptide WW291 as a template and applied sequence permutation to generate multiple candidate peptides, including Horine and Verine [30]. Despite having the same amino acid composition, these peptides exhibited distinct conformations and antimicrobial activities [30]. Horine displayed a classic amphipathic helical structure with high selectivity and systemic efficacy, while Verine demonstrated a novel conformation and broad-spectrum antimicrobial effects [30,31]. This sequence permutation strategy not only helps elucidate the relationship between peptide activity and conformation but also offers a cost-effective and predictive method for designing novel antimicrobial molecules. When combined with database analysis and high-throughput screening, this strategy becomes a powerful tool for developing new therapeutics against resistant pathogens [23,25,27]. Future success in drug development will depend on effectively integrating AI’s rapid predictive capabilities with the precise validation mechanisms of traditional methods, enabling truly intelligent drug design and clinical translation.

In our previous work, we developed the AMP W5K/A9W, which exhibited excellent antibacterial and salt-resistant properties [32,33]. To investigate the impact of lysine and tryptophan distribution, we applied a clockwise sequence permutation approach to redesign the original W5K/A9W sequence (KKWRKWLKWLAKK). This method generated a series of W5K derivatives with consistent molecular weight, net charge, and hydrophobicity. This design strategy allowed us to explore how lysine and tryptophan distribution affects peptide bioactivity. We evaluated how the distribution of these residues influences antimicrobial therapeutic index, membrane permeability, and selectivity, and used circular dichroism (CD) spectroscopy to examine their secondary structure under various conditions. Furthermore, we systematically evaluated the *in vitro* antimicrobial efficacy, salt tolerance, hemolytic potential, and wound healing properties of the designed peptides. These results enhance our understanding of the sequence–function relationship of AMPs and support their potential application in developing innovative antimicrobial therapies with wound-healing benefits.

## 2. Results

### 2.1. Sequence Permutation Design and Physicochemical Characterization of W5K Derivatives

Extensive research on antimicrobial peptides (AMPs) has revealed the role of small cationic peptides in enhancing membrane disruption and cellular penetration [13,34]. The design of AMPs is guided by various biophysical properties, including hydrophobicity, amphipathicity, secondary structure, overall net charge, size, and the balance between hydrophobic and polar regions. In this study, we based our design on the AMP W5K/A9W (KKWRKWLKWLAKK), which was derived from the previously developed AMP PEM-2 [32]. W5K/A9W exhibits potent antimicrobial activity and strong membrane-penetrating ability [33], and its helical structure has been confirmed by nuclear magnetic resonance spectroscopy [32,33].

**Figure 1:**
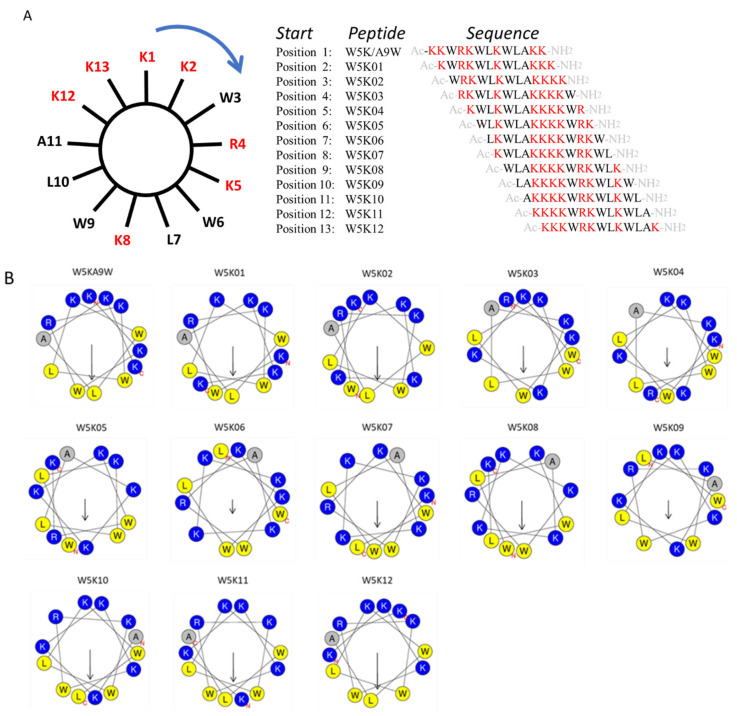
Sequence permutation generated 12 derivative peptides from W5KA9W. (**A**) The amino acid sequence of W5K/A9W is distributed evenly on the wheel. Reading from position 2 (K2) clockwise generated W5K01. Reading from position 3 (W3) in the same manner produced W5K02. Repeating this reading from position 1 to position 13 (W13) led to twelve peptides. (**B**) Helical wheel projection of W5K/A9W and its 12 derivatives. (The helical wheel was calculated and projected by the website: https://heliquest.ipmc.cnrs.fr/cgi-bin/ComputParams.py, accessed on 13 March 2023).

To investigate the impact of lysine and tryptophan distribution, we applied a clockwise sequence permutation approach to W5K/A9W (reading positions K2 to K13 in Figure 1A), resulting in 12 derivative peptides (W5K01 to W5K12, shown in Figure 1A). The helical wheel projections of the twelve W5K derivatives are illustrated in Figure 1B. With advances in bioinformatics tools, calculating the physicochemical properties of peptides via online computational platforms has become more convenient. Using the HeliQuest and ExPASy websites [35,36], we analyzed key properties of the W5K peptide family generated by sequence permutation, including W5K01 to W5K12. Parameters such as molecular weight, charge, hydrophobicity, hydrophobic moment, aliphatic index, GRAVY (grand average of hydropathy), and instability index were calculated shown in Table 1.

All W5K derivatives shared identical physicochemical properties that are independent of sequence order. They had a molecular weight of 18.4 kDa, a net charge of +7, a hydrophobicity of 0.27, an aliphatic index of 67.69, and a GRAVY value of −1.631. In contrast, the derivatives differed in hydrophobic moment and instability index, which are influenced by sequence-specific structural features. Peptides with an instability index below 40 are considered stable, and based on this criterion, all W5K derivatives listed in Table 1 are predicted to be stable. All peptides were synthesized with N-terminal acetylation and C-terminal amidation.

### 2.2. Comparative Antibacterial Activity and Salt Resistance Profiles of W5K Peptides

The antimicrobial activities of W5KA9W and its derivatives were evaluated using Gram-negative bacterium *Escherichia coli* (ATCC 25922), *Pseudomonas aeruginosa* Migula (ATCC 27853), *Acinetobacter* sp. (BCRC number 14B0091, BCRC number 14B0097, and BCRC number 14B0100), and Gram-positive bacterium *Staphylococcus aureus* sp. strains (ATCC 25923, ATCC 33591, ATCC 33592, ATCC 33593, and ATCC 6538). The results, presented in Table 2, indicate that some derivatives exhibited equal or greater bactericidal potency (equal or less than 1-fold difference) than W5KA9W in certain strains of bacteria, particularly in Gram-positive bacterium. W5K11 and W5K12 have equal or more prominent bactericidal potency (equal or less than 1-fold difference) than W5KA9W in certain strains of bacteria, especially in Gram-positive bacterium. W5K10 showed equal bactericidal potency compared to W5KA9W, except for exhibiting a 1-fold difference higher MIC value in several strains. In contrast, W5K01 and W5K02 showed 2–4 folds and 2–8 folds higher MIC values than W5KA9W, respectively, in most bacteria strains. W5K05, on the other hand, exhibited a similar level of antibacterial activity as W5K01, whereas W5K03-W5K09 demonstrated a significant loss of bactericidal potency compared to the former derivatives, with 4–16 folds higher MIC values than W5KA9W in most bacteria strains. Overall, the MIC results suggest that the polycationic lysine N-terminal fragment of the peptide sequence contributed more to the antibacterial activity than the polycationic lysine C-terminal fragment.

In addition to the testing in MH broth, the bactericidal performance of the designed peptides was evaluated in LYM broth media with varying salt concentrations (50, 100, 200, or 300 mM NaCl adjusted) and magnesium chloride concentrations (0.5, 1.5, or 2.5 mM) and pH levels (6.5, 5.5, or 4.5), as presented in Table 3, Table 4 and Table 5. In the context of varying NaCl salt concentrations, the antibacterial efficacy of W5KA9W, W5K11, and W5K12 peptides was observed to be high. However, at a salt concentration of 100 mM, the antibacterial efficacy of these peptides began to weaken. Specifically, at a concentration of 300 mM NaCl, the antibacterial efficacy of W5KA9W and W5K12 peptides was found to be weakened by up to 4-fold, while the efficacy of W5K11 peptide was weakened by up to 3-fold. Despite being slightly weaker in antibacterial efficacy compared to the high-efficiency antibacterial peptides mentioned above, the W5K10 peptide exhibited similar characteristics to W5KA9W and W5K12 peptides. Its bactericidal efficacy began to be affected at a concentration of 100 mM NaCl, and in the concentration environment, its efficacy was weakened by up to 4-fold. The W5K01 and W5K02 peptides demonstrated similar behavior, with their antibacterial efficacy beginning to weaken at increasing NaCl salt concentrations. Under the environment of 200 mM NaCl salt concentration, these peptides essentially lost their bactericidal efficacy against the tested strains. Similarly to the W5K01 and W5K02 peptides, the bactericidal efficacy of the W5K03-W5K09 peptides also began to weaken as the NaCl salt concentration increased, with these peptides losing their bactericidal effect on the test strain under the environment of 100 mM NaCl salt concentration (Table 3 and Figure S1).

In the context of varying MgCl_2_ salt concentrations, the antibacterial efficacy of W5KA9W, W5K11, and W5K12 peptides remained high up to a concentration of 0.5 mM (Table 4). However, as the MgCl_2_ salt concentration continued to rise, the antibacterial efficacy of these peptides was observed to weaken by up to 2-fold. In contrast, the W5K10 peptide showed good antibacterial efficacy, with its bactericidal effect beginning to be affected at a salt concentration of 1.5 mM MgCl_2_. The W5K01 and W5K02 peptides also demonstrated decreasing antibacterial efficacy as the MgCl_2_ salt concentration increased, with their bactericidal efficacy being weakened by 4-fold under the environment of 2.5 mM MgCl_2_ salt concentration. The W5K03-W5K09 peptides exhibited similar behavior, with their antibacterial efficacy beginning to weaken in the environment of 0.5 mM MgCl_2_ salt concentration (Table 4 and Figure S2). Notably, under the pH-adjusted environment, the designed peptides exhibited the same bactericidal efficacy as the initial neutral environment (pH 7.4) (Table 5 and Figure S3). Our findings indicate that adjusting the NaCl salt concentration had the greatest impact on the antibacterial activity of W5KA9W and its derivatives under the test conditions.

Overall, the results of the antibacterial and salt resistance tests suggest that the N-terminal sequences of polycationic lysine W5K11 and W5K12 demonstrated superior antibacterial and NaCl salt-resistant ability compared to the other designed peptides. Additionally, the W5K10 peptide, with the addition of an alanine amino acid to the N-terminal sequence of polycationic lysine, exhibited promising antibacterial and salt resistant effects.

### 2.3. Hemolytic Activity and Membrane Selectivity of the Designed Peptides

**Figure 2:**
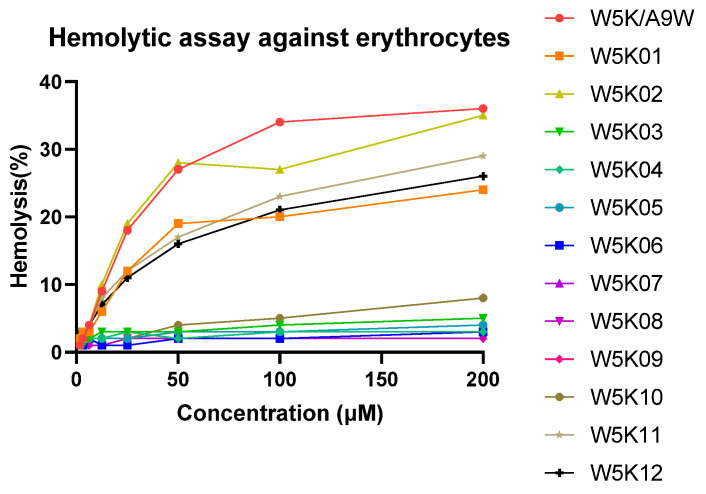
Hemolytic activities of W5K/A9W and its derivatives. Equal volumes of peptides and hRBCs were mixed and incubated at 37 °C for 1 h. The supernatant was collected from the samples and tested for absorbance at 565 nm. Zero hemolysis (blank) was defined in PBS buffer, while 100% hemolysis was defined in 1% Triton X-100.

The hemolytic effects of W5KA9W and its 12 derivatives on human red blood cells (RBCs) were depicted in Figure 2, with the RBCs being lysed in the following order: W5KA9W ≈ W5K02 > W5K12 > W5K11 ≈ W5K01 >> W5K10 > other W5K derivatives. Notably, the N-terminal serial lysine residues of W5K11 and W5K12, and the C-terminal serial lysine residues of W5K01 exhibited lower hemolytic activity compared to the original W5KA9W sequence, except for W5K02. Interestingly, while exposure to W5KA9W, W5K02, W5K12, W5K11, and W5K01 resulted in the lysis of approximately 35%, 34%, 30%, 27%, and 25% of RBCs, respectively, less than 10% of RBCs were lysed after exposure to W5K10 at a concentration of 200 μM. The strong hemolytic activity observed for the W5K series peptides with strong antibacterial activity is consistent with findings from previous literature. However, it is noteworthy that W5K10 displayed a significant reduction in hemolytic activity while maintaining a certain level of bactericidal activity, which was unexpected.

### 2.4. Evaluation of Therapeutic Index: Balancing Antibacterial Potency and Cytotoxicity

In this study, we aimed to investigate the potential of W5K series peptides for therapeutic use. To evaluate their efficacy for antibacterial therapy, we used the Therapeutic Index (TI) as a measure of their cell selectivity [37,38]. The TI was calculated as the ratio of the concentration causing 10% hemolysis of human red blood cells (HC_10_) to the geometric mean minimum inhibitory concentration (GM) of the peptides against both Gram-negative and Gram-positive bacteria. This parameter was used to quantitatively assess the balance between antibacterial potency and cytotoxicity among the W5K derivatives.

Table 6 presents the TI values of various W5K derivatives against Gram-negative bacteria, Gram-positive bacteria, and their combination. It is evident that W5K10 and W5K11 exhibit significantly higher TI values than the other W5K derivatives, including the original sequence W5K/A9W. W5K11 has a high TI value owing to its potent antibacterial activity and low hemolytic activity compared to W5K/A9W. Similarly, W5K10 has a lower hemolytic activity and retains a certain level of antibacterial activity, which contributes to its high TI value. These findings demonstrate the potential of W5K series peptides, particularly W5K10 and W5K11, as promising candidates for antibacterial therapy.

### 2.5. In Vitro Wound-Healing Activity of W5K Peptides in Keratinocyte Migration

**Figure 3:**
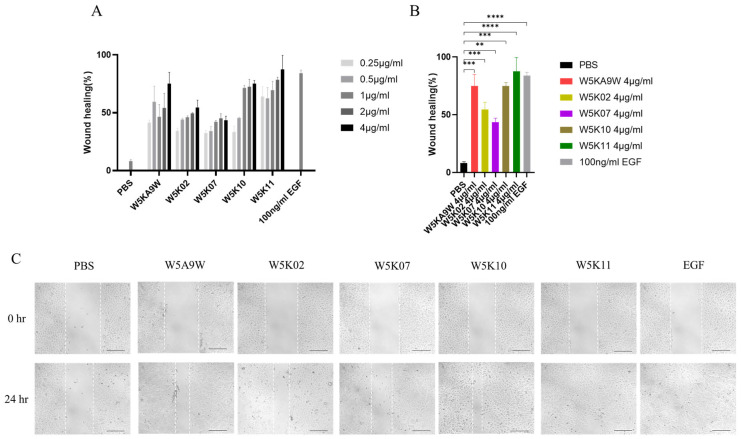
Cell migration rate (*In Vitro* wound healing rate) of HaCaT cells induced by the designed peptides. (**A**) Bars shows the percentage of cell migration rate of W5K/A9W and its derivates from 0.25 µg/mL to 4 µg/mL for culture 24 h. (**B**) Bars shows the percentage of cell migration rate of 4 µg/mL W5K/A9W and its derivates for culture 24 h. (**C**) Images show the cell migration of 4 µg/mL W5K/A9W and its derivates for culture 24 h. (Bar: 200 μm) Results are presented as means ± standard deviations (SD); *n* = 3 (three independent experiments), ns = no significant differences; ** *p* < 0.01; *** *p* < 0.001; **** *p* < 0.0001 compared with PBS (Negative Control) and 100 ng/mL EGF (Positive Control).

Regarding their potential therapeutic use, antibacterial peptides are frequently utilized as active ingredients in topical medications [10,39,40]. As such, we conducted an investigation to evaluate the effectiveness of W5K/A9W and its derivatives *in vitro* for wound healing purposes. Figure 3 displays the results of the *in vitro* wound healing assay performed on immortalized keratinocyte cell lines (HaCaT) treated with the W5K/A9W peptide and its derivatives. The cell migration rate for the different concentrations of W5K/A9W and its derivatives after 24 h is presented in Figure 3A. The findings indicate that all of the peptides tested had a beneficial wound healing effect at various concentrations, exhibiting dose-dependent behavior compared to the negative control group. Notably, W5K11 demonstrated a superior wound healing effect even at the lowest concentration of 0.25 µg/mL. Additionally, the results for the peptides treated with a concentration of 4 µg/mL were combined in Figure 3B,C. The findings suggest that W5K/A9W, W5K10, and W5K11 have comparable wound healing effects.

### 2.6. Peptide-Induced Membrane Permeabilization in Model Liposomes

**Figure 4:**
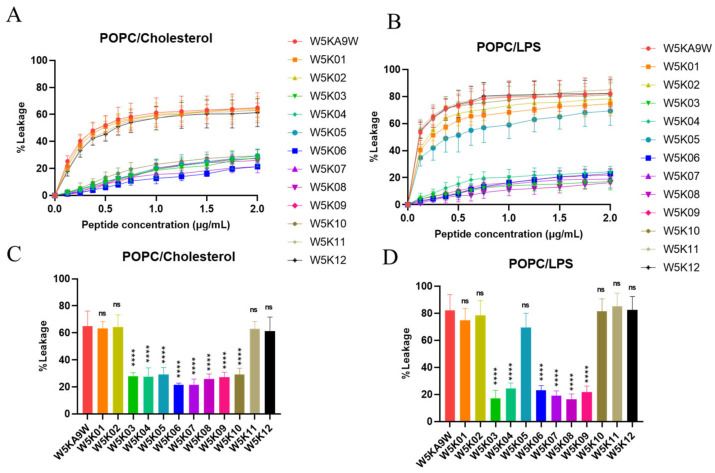
Membrane permeabilization of LUVs induced by designed peptides. Plot shows the percentage of calcein leakage of W5K/A9W and its 12 derivates in (**A**) POPC:Cholesterol LUVs and (**B**) POPC:LPS LUVs. Results are presented as means ± standard deviations (SD); *n* = 3 (three independent experiments). Bars shows the percentage of calcein leakage of 2 µg/mL W5K/A9W and its 12 derivates in (**C**) POPC:Cholesterol LUVs and (**D**) POPC:LPS LUVs. Results are presented as means ± standard deviations (SD); *n* = 3 (three independent experiments), ns = no significant differences; **** *p* < 0.0001 compared with W5K/A9W.

In this experiment, we investigated the efficacy of designed peptides in disrupting the membranes of different types of phospholipid vesicles. To simulate the surface charges of different biological cell membranes, we utilized artificial phospholipid vesicles. Our approach involved exploring the ability of the peptides to permeabilize these vesicles by measuring the release degree of calcein. We created three types of phospholipid vesicles, including POPC:Cholesterol LUVs to mimic eukaryotic cell membranes and POPC:LPS LUVs to mimic lipopolysaccharide-containing membranes on Gram-negative bacterial membranes. The experimental results of peptide-induced permeabilization of LUVs are shown in Figure 4.

Our results indicate that, among the POPC:Cholesterol LUVs, sequences from W5K03 to W5K10 exhibit weak membrane disrupting activity. Meanwhile, W5K/A9W, W5K01, W5K02, W5K11, and W5K12, which contain poly lysine concentrated in the N-terminal or C-terminal design peptides, show strong membrane disrupting activity. These peptides at a final concentration of 2 μg/mL exhibit leakage above 60% (Figure 4A,C). Designed peptides in POPC: Cholesterol LUVs liposome permeation was similar to the hemolytic activity curve (Figure 2). Similarly, in POPC:LPS LUVs, W5K/A9W, W5K01, W5K02, W5K11, and W5K12 exhibit strong membrane-damaging activity, with respective leakages of 82%, 74%, 78%, 85%, and 82% at a final concentration of 2 μg/mL. Surprisingly, W5K05 and W5K10 also show high leakage rates of 69% and 81% at a final concentration of 2 μg/mL. Other designed peptides only cause less than 25% leakage in POPC:LPS LUVs (Figure 4B,D). The permeabilization of liposomes with designed peptides in POPC:LPS LUVs is similar to that against Gram-negative bacteria (Table 2).

These results demonstrate the selectivity of the designed peptides for different species of biomembranes, and their activity is largely related to the cell membrane charge. While W5K/A9W, W5K01, W5K02, W5K11, and W5K12 exhibit better membrane permeabilization activity, they have strong penetration effects on all charged LUVs, and their membrane selectivity is not good. While, surprisingly, W5K10 exhibits good membrane permeabilization activity and membrane selectivity. Our findings suggest that the distribution of poly lysine affects membrane permeabilization activity. Both the N-terminal and C-terminal of the poly lysine distribution sequence result in a peptide with high membrane permeabilization activity, but the membrane selectivity of the peptide may be lost. Surprisingly, adding alanine in front of the poly lysine sequence displayed by the W5K10 peptide, led to enhanced membrane selectivity. This finding partly justifies the need for further investigation into sequence properties, which we are currently conducting follow-up research in this area. Based on the current experimental results, it is evident that the sequence of multiple positive charges can influence the membrane permeabilization activity. As such, careful consideration of the positive charge distribution is necessary when designing antimicrobial peptides.

### 2.7. Secondary Structure Analysis of W5K Peptides by Circular Dichroism (CD) Spectroscopy

**Figure 5:**
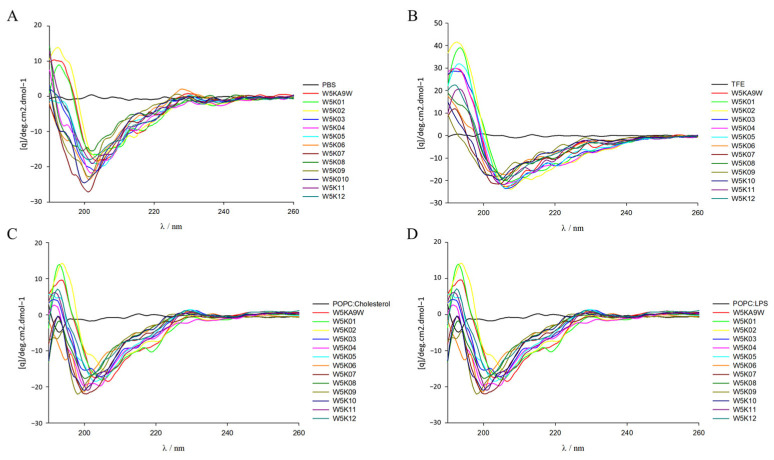
Circular dichroism spectra of W5K/A9W and its 12 derivates. CD spectra were recorded at 60 μM concentration of W5K/A9W and its derivates in (**A**) 20 mM phosphate buffer, (**B**) 30% TFE buffer, (**C**) 1 mM POPC:Cholesterol LUVs and (**D**) 1 mM POPC:LPS LUVs at pH7.4, 25 °C.

In the design of antimicrobial peptides, the secondary structure is a key consideration, and CD spectroscopy is commonly used to examine the configuration differences of designed peptides in various environments and explore their secondary structure. As shown in Figure 5C,D spectra of the designed peptides were obtained under different conditions, including PBS, 30% TFE, POPC/Cholesterol and POPC/LPS. The designed peptide displays a random coil conformation in the spectra of PBS aqueous solution and POPC/Cholesterol and other electrically neutral environments, as shown in Figure 5A,C. However, in the presence of TFE, increasingly stronger negative bands were observed at 209 and 220 nm, which suggests the possible formation of a helical structure of the designed peptides in a negatively charged environment (Figure 5B). Conversely, in the case of POPC/LPS, the designed peptides tend to exhibit a random coil conformation in spectra, as shown in Figure 5D. The observed lack of consistency between the helicity of the peptides in the model membrane and their antibacterial, and hemolytic activity, is intriguing. The structure of the W5K/A9W sequence was previously solved using NMR spectroscopy, revealing an α-helical structure for the middle 9 amino acids (WRKWLKWLA), while the head-to-tail double repeat lysine displayed a random coil conformation. Thus, in this experiment, the secondary structure of the designed peptides is likely to be poly lysine at the head and tail of the sequence and within the sequence, which may have affected the detection of the overall series of helical structures by CD spectrum. In the future, NMR spectroscopy may be considered to examine the information related to the structure of the designed peptide and then explore the relationship between activity and structure. It is noteworthy that the peptides with strong membrane permeabilization activity, namely W5K/A9W, W5K01, W5K02, W5K11, and W5K12, and the W5K10 peptide, which exhibited favorable membrane permeabilization activity and membrane selectivity, did not demonstrate a proportional increase in their helix calculations as compared to the less active peptides (Table 7). Overall, these results suggest that basic secondary structure testing methods may not be suitable for determining the structural properties of this series’ antimicrobial peptides and as such may not serve as reference parameters for future design of such peptides.

## 3. Discussion

This study systematically explored the effect of the distribution of lysine and tryptophan in antimicrobial peptide sequences on their functional properties. We found that when polylysine sequences (such as KKKK) are positioned at the N-terminus, the antibacterial activity against Gram-positive and Gram-negative bacteria can be significantly enhanced, while the hemolytic toxicity to human erythrocytes can be effectively reduced. This arrangement strategy further improves the therapeutic index (TI = MIC/HC_10_), which reflects the balance between antibacterial efficacy and host cell safety by comparing peptide hemolytic concentration with MIC, and has been widely used as a standard measure for evaluating AMP selectivity. Balancing antimicrobial efficacy and host cell safety remains a major challenge for AMP clinical application [41,42], highlighting the importance of TI optimization [43,44,45,46].

We verified through systematic linear sequence permutation experiments that even when the amino acid composition is exactly the same, simply adjusting the order of the amino acids can significantly change the therapeutic index and membrane selectivity of AMP. We designed 12 different linear variants using the 13-amino acid W5K/A9W as the parent sequence and found that they showed significant differences in antibacterial and hemolytic activities. Among them, W5K11 (polylysine positioned at the N-terminus) exhibited the highest therapeutic index, indicating that it possesses the ideal properties of high antibacterial efficacy and low host toxicity. We speculate that this arrangement design helps to strengthen the electrostatic interaction with negatively charged components on the bacterial membrane (such as lipopolysaccharide and phosphatidic acid), while reducing nonspecific binding to cholesterol-rich, neutrally charged mammalian cell membranes, thereby improving membrane selectivity and reducing side effects.

In contrast, when the KKKK polylysine sequence was moved to the C-terminus or the middle segment, an increase in hemolytic toxicity and a decrease in therapeutic index were observed, further supporting the importance of “N-terminal polypositive design” in enhancing the safety and selectivity of AMPs. In addition, adding a neutral amino acid (such as alanine, see W5K10) before the N-terminal polylysine sequence can also effectively reduce hemolytic toxicity without compromising antibacterial activity, providing a simple but effective low-toxicity design strategy. These results show that even very slight changes in sequence arrangement can cause significant functional differences, highlighting the feasibility and practicality of the design strategy of “maximizing the therapeutic index” in AMP engineering.

Our results are very similar to those of previous studies on the WW291 series of antimicrobial peptides, further confirming the key influence of sequence arrangement on AMP activity and selectivity [30,31]. Wang et al. pointed out that the broad-spectrum antimicrobial efficacy of WW295 was attributed to its atypical α-helical structure and the vertical separation of hydrophilic/hydrophobic regions [30,31]. Similar phenomena were also observed in our W5K series, especially when polylysine was positioned at the N-terminus, which helped to form a vertical amphiphilic structure, strengthen the electrostatic interaction with the negatively charged components of the bacterial membrane, and reduce the nonspecific binding to the mammalian cell membrane, thereby improving the antibacterial selectivity and therapeutic index. In particular, the clustering of poly-lysine residues at the N-terminus may induce an axial polarization of positive charges along the helix, generating a “vertical spiral” topology distinct from the conventional horizontal amphiphilicity depicted by two-dimensional helical wheel models. Such a three-dimensional charge distribution could enhance directional electrostatic attraction toward negatively charged bacterial membranes and explain the observed differences in selectivity and therapeutic index. When peptide molecules have clear hydrophilic and hydrophobic partitions, especially through N-terminal polycation design, they are more likely to insert into bacterial membranes rather than host cell membranes, further supporting our observations on the association between sequence arrangement and function.

Calcein release assays showed that peptides with distinct hydrophilic/hydrophobic segregation and N-terminal lysine clustering (such as W5K11 and W5K12) exhibited strong disruptive effects on both bacterial and host cell membranes, reflecting their high membrane-penetrating capability. In contrast, W5K10 caused significant disruption of bacterial membranes but relatively low effects on host membranes, indicating a degree of selectivity in its membrane-disruptive behavior. These results support our hypothesis that sequence arrangement influences AMP activity and selectivity: the position of poly-lysine within the sequence modulates the amphipathic distribution and helical orientation of the peptide, thereby affecting its membrane interactions and penetration depth. Different sequence arrangements not only determine the strength of membrane disruption but also directly impact selectivity toward bacterial versus host cell membranes, providing important structural insights for the design of highly selective antimicrobial peptides.

In addition to antimicrobial activity, we also observed that several W5K-derived peptides (especially W5K10 and W5K11) have the potential to promote cell migration and accelerate wound healing, which is similar to the known immunomodulatory, angiogenesis and tissue repair functions of various natural AMPs (such as LL-37, HBD3, IDR-1018 and PR-39) [40,47,48,49,50,51]. While our study utilized a cell-based model for initial screening, future work will employ full-thickness murine wound models under both infected and non-infected conditions to evaluate the dual effects of these peptides. These studies will assess not only antimicrobial activity but also tissue responses such as inflammation and regeneration, providing a more comprehensive understanding of whether the sequence-dependent selectivity and membrane-targeting properties observed *in vitro* translate into effective in vivo outcomes.

Although previous studies have shown that most AMPs tend to form α-helical structures in negatively charged environments [52,53,54], our circular dichroism (CD) analysis showed that there is no clear correspondence between the helical content of the W5K series and its antibacterial or hemolytic function. Our previous nuclear magnetic resonance (NMR) studies of W5K/A9W also showed that its central segment forms an α-helix, while the two lysine-rich ends are unstructured [32,33]. This result emphasizes that the relationship between the structure and function of AMPs is more complex than expected, and high-resolution techniques will still be required to further elucidate their mechanism of action. We have already initiated NMR-based structural studies to characterize the true three-dimensional conformations of the W5K series peptides. In addition, future studies will employ computational approaches, including molecular docking and molecular dynamics simulations, to investigate the interactions between these peptides and hemolysis-related membrane proteins or lipid components, providing deeper insights into their membrane-permeation and hemolytic mechanisms.

Together, our findings support a sequence permutation-based AMP optimization strategy, emphasizing the importance of charge and hydrophobic residue distribution in governing bioactivity and selectivity. Even minor rearrangements within short peptide sequences can result in substantial functional differences, enabling precision engineering of AMPs. In the future, the integration of AI-driven predictive models with sequence permutation datasets may facilitate efficient, high-throughput, and cost-effective design of antimicrobial peptides. Given the growing global challenge of antibiotic resistance, this strategy holds strong potential for the development of multifunctional and safer peptide therapeutics for bacterial infections and chronic wound management.

## 4. Materials and Methods

### 4.1. Materials

All peptides were obtained from Kelowna Int’l Scientific Inc. (New Taipei City, Taiwan) and their identity was confirmed using matrix-assisted laser desorption/ionization time-of-flight/time-of-flight (MALDI-TOF/TOF) mass spectrometry (Autoflex III, Bruker Daltonics, Leipzig, Germany).. Purity (>95%) was assessed using the Waters 2796 BioSeparations Module HPLC (Waters, Milford, MA, USA). Müller-Hinton broth (MHB) was purchased from Becton, Dickinson and Company (Franklin Lakes, NJ, USA). Sigma-Aldrich (St. Louis, MO, USA) was the source for lipopolysaccharides from Escherichia coli O26:B6, cholesterol, and calcein. Avanti Polar Lipids, Inc. (Alabaster, AL, USA) provided POPC lipids.

### 4.2. Bacterial Strains and Culture Conditions

The antibacterial and salt-resistant activity of the peptides was tested using *Escherichia coli* (ATCC 25922), *Pseudomonas aeruginosa* Migula (ATCC 27853), Acinetobacter sp. (BCRC number 14B0091, BCRC number 14B0097, and BCRC number 14B0100), and *Staphylococcus aureus* sp. strains (ATCC 25923, ATCC 33591, ATCC 33592, ATCC 33593, and ATCC 6538). All bacteria were obtained from the Food Industry Research and Development Institute (Hsinchu, Taiwan) and cultured in sterilized Müller-Hinton (MH) broth at 37 °C and 200 rpm for 8 h. The inoculum concentrations were determined by measuring the optical density at 600 nm (OD 600 = 1, approximately 10^8^ CFU/mL) using an Ultrospec 2100 pro UV-Visible spectrophotometer (Biochrom Ltd., Cambridge, UK) after 8 h of culture.

### 4.3. Antimicrobial and Salt Resistant Activity Assay

The antibacterial activities of the peptides in Müller-Hinton (MH) broth and modified LYM broth were determined using the standard broth microdilution method as per Clinical and Laboratory Standards Institute (CLSI) guidelines [55]. The LYM broth was prepared with specific components and adjusted to different conditions, including varying concentrations of NaCl and MgCl_2_, and different pH levels. To prepare the LYM broth, 5.4 mM KCl, 5.6 mM Na_2_HPO_4_, 0.5 mM MgSO_4_, and 1.0 mM sodium citrate were added. Additionally, 0.4 mg of ZnCl_2_, 2.0 mg of FeCl_3_·6H_2_O, 0.1 mg of CuSO_4_·5H_2_O, 0.1 mg of MnSO_4_·H_2_O, 0.1 mg of Na_2_B_4_O_7_∙10H_2_O, 700 mg of amino acid mixtures without tryptophan (Takara Bio Inc., San Jose, CA, USA), and 20 mg of L-tryptophan were added per liter of medium. The pH was adjusted to 7.4, and a vitamin mixture (100X, Sigma) and glucose concentration of 2% were added. For different conditions of LYM broth, NaCl concentrations of 300, 200, 150, and 50 mM, MgCl_2_ concentrations of 2.5, 1.5, and 0.5 mM, and pH levels of 6.5, 5.5, and 4.5 were adjusted. Bacterial strains were incubated in MH broth overnight, regrown, and diluted to a final concentration of 5 × 10^5^ CFU/mL. Peptides were added to each well at final concentrations of 50, 25, 12.5, 6.25, 3.13, 1.56, and 0.78 µg/mL, and 99 µL of diluted microbes was loaded into each well of a polypropylene 96-well plate. The minimum inhibitory concentration (MIC) was defined as the lowest peptide concentration that inhibited 90% of visible bacterial growth after 16 h of incubation at 37 °C. The Morpheus HeatMap software (https://software.broadinstitute.org/morpheus/) was used to display MIC values on a color scale [56]. All experiments were independently repeated three times.

### 4.4. Hemolytic Activity Assay

Hemolytic activity was evaluated by testing the ability of the peptides to cause lysis of human red blood cells (hRBCs), both in the presence and absence of the antimicrobial peptide. Blood samples were collected from healthy volunteers and treated with ethylenediaminetetraacetic acid (EDTA) to prevent coagulation. The hRBCs were then washed three times with phosphate-buffered saline (PBS) and pelleted by centrifugation at 800× *g* for 10 min. Next, the hRBCs were diluted in PBS to a final concentration of 10%. Peptides were serially diluted in PBS ranging from 400 µM to 3.13 µM, and an equal volume of 10% hRBC was added to each dilution. After incubation at 37 °C for 1 h, the supernatant was collected by centrifugation at 800× *g* for 10 min, and the absorbance at 565 nm was measured. Negative controls (PBS blank) and positive controls (1% Triton X-100 in PBS) were also included. The percentage of hemolysis was calculated using the following formula:

%hemolysis = [(A_sample_ − A_PBS_)/(A_TritonX-100_ − A_PBS_)] × 100%,

where A is the absorbance under the specified conditions.

### 4.5. Wound Healing Assay

For the Cell Migration Assay (*in vitro* wound healing assay), HaCaT cells (4 × 10^4^ cells) were seeded on each side of an ibidi culture insert (ibidi, Germany) in DMEM medium supplemented with 10% fetal bovine serum. The inserts were placed into a 24-well plate and incubated at 37 °C and 5% CO_2_ overnight. After removing the inserts, 1 mL of serum-free DMEM medium supplemented with different concentrations of peptide was added. Medium without peptide and with 100 ng/mL EGF (epidermal growth factor) were used as negative and positive controls, respectively. The migration area was imaged at 0 and 24 h using an inverted fluorescent microscope (Zeiss/Observer Z1) at 10× magnification, and the area was quantified using ImageJ software(version 1.54g). The repairing rate was calculated using the formula:

Repairing Rate = (Area_0h_-Area_24h_)/Area_0h_,

where 0 h and 24 h represent the time points at which the images were taken.

### 4.6. Preparation of Large Unilamellar Vesicles (LUVs)

Large unilamellar vesicles (LUVs) containing POPC:LPS (12.5:1, mol/mol) and POPC:cholesterol (2:1, mol/mol) were prepared using the extrusion method and an Avanti small-volume extrusion apparatus(Avanti Polar Lipids) [57,58]. To prepare the vesicles, phospholipids were dissolved in a chloroform/methanol mixture (4:1, *v*/*v*), evaporated using nitrogen gas, and suspended in PBS buffer. The lipid suspensions underwent six to eight cycles of freezing and thawing before being extruded ten times through a 0.4 mm pore size polycarbonate filter, followed by ten times through a 0.1 mm filter. Calcein-filled LUVs were also prepared by loading calcein-containing buffer (70 mM calcein and 10 mM Tris at pH 7.4) into a Sephadex G-75 column loaded with iso-osmotic buffer (100 mM sodium chloride and 10 mM Tris) to remove unencapsulated calcein. The phospholipid content of the vesicles was determined by assessing inorganic phosphate using the ammonium ferrothiocyanate method described by John Charles Marshall Stewart [59].

### 4.7. Dye Leakage Assay

Calcein leakage induced by peptides was measured using a Perkin-Elmer luminescence spectrofluorimeter with excitation and emission wavelengths of 496 and 515 nm, respectively. Calcein-entrapped LUVs were diluted to a concentration of 10 µM. The maximum degree of leakage was induced by 100 mg/mL Triton X-100.

The degree of leakage induced by various peptide concentrations was calculated using the formula:

Leakage % = ((F − F0)/(Fr − F0)) × 100%,

where F0 and Fr represent the initial fluorescence intensities observed without peptide and after adding 100 mg/mL Triton X-100, respectively.

### 4.8. Circular Dichroism (CD) Spectroscopy

CD spectra were recorded on an AVIV 202 spectropolarimeter after calibration with d-l0-camphorsulfonic acid. The measurements were performed using a 1 mm path-length cuvette with 20 mM phosphate buffer, TFE, and vesicles of POPC/cholesterol and POPC/LPS, with a scanned wavelength range of 190–260 nm at 25 °C. Peptide and liposome concentrations were adjusted to 60 mM and 1 mM, respectively, using 20 mM phosphate buffer at pH 7.4. Each spectrum was an average of three scans with a 0.2 nm step size. The data were corrected using appropriate baselines and converted to molar ellipticity (degree cm^2^ dmol^−1^).

### 4.9. Statistical Analysis

The statistical results are presented as the mean ± standard deviation (SD) and were analyzed using one-way analysis of variance (ANOVA). GraphPad Prism version 8.0 (San Diego, CA, USA) was used for statistical analysis, and p values less than 0.05 were considered to indicate a statistically significant difference.

## 5. Conclusions

This study demonstrates that the spatial arrangement of lysine and tryptophan residues within the AMP sequence plays a decisive role in regulating antimicrobial potency, cell selectivity, and hemolytic activity. Specifically, positioning poly-lysine at the N-terminus, with optional insertion of alanine residues, provides an effective strategy to enhance therapeutic potential and reduce toxicity. The use of sequence permutations provides a rational and efficient framework for AMP optimization, enabling the generation of multiple peptide variants with customized properties. Furthermore, the discovery of wound healing potential in W5K derivatives expands the application of these peptides beyond infection control. Looking to the future, combining AI-guided predictive models with sequence alignment strategies and high-throughput screening may accelerate the development of next-generation AMPs. In an era of increasing antibiotic resistance, this approach offers a promising avenue for versatile, safe, and clinically translatable antimicrobial agents.

## Figures and Tables

**Table 1:** The sequences alignment and physical properties of W5K/A9W and its other 12 derivatives.

Name	Sequence	<μH> ^a^	Instability Index ^b^
W5K/A9W	Ac-KKWRKWLKWLAKK-NH2	0.72	21.68
W5K01	Ac-KWRKWLKWLAKKK-NH2	0.59	21.68
W5K02	Ac-WRKWLKWLAKKKK-NH2	0.67	21.68
W5K03	Ac-RKWLKWLAKKKKW-NH2	0.54	21.68
W5K04	Ac-KWLKWLAKKKKWR-NH2	0.39	21.68
W5K05	Ac-WLKWLAKKKKWRK-NH2	0.44	21.68
W5K06	Ac-LKWLAKKKKWRKW-NH2	0.28	12.19
W5K07	Ac-KWLAKKKKWRKWL-NH2	0.52	28.22
W5K08	Ac-WLAKKKKWRKWLK-NH2	0.57	21.68
W5K09	Ac-LAKKKKWRKWLKW-NH2	0.37	12.19
W5K10	Ac-AKKKKWRKWLKWL-NH2	0.61	21.68
W5K11	Ac-KKKKWRKWLKWLA-NH2	0.61	21.68
W5K12	Ac-KKKWRKWLKWLAK-NH2	0.75	21.68

^a^ Calculated from heliquest (https://heliquest.ipmc.cnrs.fr/cgi-bin/ComputParamsV2.py, accessed on 13 March 2023). <H>: Hydrophobicity; <μH>: Hydrophobic moment. ^b^ Calculated from ExPASy (https://web.expasy.org/protparam/, accessed on 13 March 2023).

**Table 2:** MIC (μg/mL) values for W5K/A9W and its derivatives.

Bacterial Strains	MIC (μg/mL)												
	W5K/A9W	W5K01	W5K02	W5K03	W5K04	W5K05	W5K06	W5K07	W5K08	W5K09	W5K10	W5K11	W5K12
*E. coli* ATCC 25922	3.13	12.5	12.5	50	25	12.5	25	50	50	25	6.25	6.25	**3.13**
*P. aergionsa* ATCC 27853	3.13	12.5	25	50	50	25	50	50	25	25	6.25	**3.13**	**3.13**
*A. baumannii* 14B0091	1.56	6.25	3.13	6.25	12.5	12.5	50	12.5	>50	50	**1.56**	**0.78**	**1.56**
*A. baumannii* 14B0097	1.56	6.25	3.13	6.25	12.5	6.25	50	12.5	25	25	**1.56**	**1.56**	**1.56**
*A. baumannii* 14B00100	1.56	6.25	6.25	12.5	12.5	12.5	50	12.5	>50	50	3.13	**1.56**	3.13
*S. aureus* ATCC 25923	3.13	12.5	25	50	50	25	50	50	25	25	6.25	**3.13**	**3.13**
*S. aureus* ATCC 33591	12.5	50	25	>50	>50	50	>50	>50	>50	>50	**12.5**	**12.5**	**6.25**
*S. aureus* ATCC 33592	12.5	50	25	>50	>50	50	>50	>50	>50	>50	**12.5**	**6.25**	**6.25**
*S. aureus* ATCC 33593	12.5	50	25	>50	>50	50	>50	>50	>50	>50	**12.5**	**6.25**	**6.25**
*S. aureus* ATCC 6538	6.25	12.5	12.5	25	25	12.5	50	>50	>50	>50	**6.25**	**3.13**	**3.13**

Peptides showing MIC values equal to or lower than those of the parent peptide W5K/A9W are highlighted in bold.

**Table 3:** MIC (μg/mL) values for W5KA9W and its derivatives in different concentrations of NaCl.

	MIC (μg/mL)												
Bacterial strains	MH												
	W5K/A9W	W5K01	W5K02	W5K03	W5K04	W5K05	W5K06	W5K07	W5K08	W5K09	W5K10	W5K11	W5K12
*E. coli* ATCC 25922	3.13	12.5	12.5	50	25	12.5	25	50	50	25	6.25	6.25	**3.13**
*P. aergionsa* ATCC 27853	3.13	12.5	25	50	50	25	50	50	25	25	6.25	**3.13**	**3.13**
*A. baumannii* 14B0091	1.56	6.25	3.13	6.25	12.5	12.5	50	12.5	>50	50	**1.56**	**0.78**	**1.56**
*A. baumannii* 14B0097	1.56	6.25	3.13	6.25	12.5	6.25	50	12.5	25	25	**1.56**	**1.56**	**1.56**
*A. baumannii* 14B00100	1.56	6.25	6.25	12.5	12.5	12.5	50	12.5	>50	50	3.13	**1.56**	3.13
*S. aureus* ATCC 25923	3.13	12.5	25	50	50	25	50	50	25	25	6.25	**3.13**	**3.13**
*S. aureus* ATCC 33591	12.5	50	25	>50	>50	50	>50	>50	>50	>50	**12.5**	**12.5**	**6.25**
*S. aureus* ATCC 33592	12.5	50	25	>50	>50	50	>50	>50	>50	>50	**12.5**	**6.25**	**6.25**
*S. aureus* ATCC 33593	12.5	50	25	>50	>50	50	>50	>50	>50	>50	**12.5**	**6.25**	**6.25**
*S. aureus* ATCC 6538	6.25	12.5	12.5	25	25	12.5	50	>50	>50	>50	**6.25**	**3.13**	**3.13**
Bacterial strains	0 mM NaCl												
	W5K/A9W	W5K01	W5K02	W5K03	W5K04	W5K05	W5K06	W5K07	W5K08	W5K09	W5K10	W5K11	W5K12
*E. coli* ATCC 25922	1.56	3.13	6.25	6.25	6.25	**1.56**	6.25	12.5	3.13	6.25	3.13	**1.56**	**1.56**
*P. aergionsa* ATCC 27853	1.56	3.13	6.25	6.25	12.5	3.13	6.25	6.25	3.13	3.13	3.13	**1.56**	**1.56**
*A. baumannii* 14B0091	1.56	**1.56**	**1.56**	**1.56**	**1.56**	**1.56**	3.13	3.13	3.13	**1.56**	**1.56**	**1.56**	**1.56**
*A. baumannii* 14B0097	1.56	**1.56**	**1.56**	**1.56**	**1.56**	**1.56**	3.13	3.13	3.13	3.13	**1.56**	**1.56**	**1.56**
*A. baumannii* 14B00100	1.56	**1.56**	**1.56**	**1.56**	**1.56**	**0.78**	3.13	3.13	3.13	3.13	**1.56**	**0.78**	**1.56**
*S. aureus* ATCC 25923	1.56	3.13	6.25	6.25	6.25	**1.56**	6.25	6.25	**1.56**	3.13	3.13	**1.56**	**1.56**
*S. aureus* ATCC 33591	3.13	6.25	6.25	25	25	6.25	12.5	25	25	25	**3.13**	**3.13**	**3.13**
*S. aureus* ATCC 33592	3.13	6.25	6.25	25	25	6.25	12.5	25	25	25	**3.13**	**3.13**	**3.13**
*S. aureus* ATCC 33593	3.13	6.25	6.25	25	25	6.25	12.5	25	25	25	**3.13**	**3.13**	**3.13**
*S. aureus* ATCC 6538	1.56	3.13	6.25	**1.56**	3.13	3.13	6.25	6.25	12.5	6.25	**1.56**	**1.56**	**1.56**
Bacterial strains	50 mM NaCl												
	W5K/A9W	W5K01	W5K02	W5K03	W5K04	W5K05	W5K06	W5K07	W5K08	W5K09	W5K10	W5K11	W5K12
*E. coli* ATCC 25922	1.56	6.25	12.5	25	25	3.13	12.5	25	12.5	12.5	3.13	**1.56**	3.13
*P. aergionsa* ATCC 27853	1.56	6.25	6.25	12.5	12.5	6.25	6.205	12.5	6.25	6.25	6.25	**1.56**	**1.56**
*A. baumannii* 14B0091	1.56	3.13	**1.56**	3.13	3.13	**1.56**	3.13	6.25	6.25	6.25	**1.56**	**1.56**	**1.56**
*A. baumannii* 14B0097	1.56	**1.56**	**1.56**	3.13	3.13	3.13	3.13	6.25	6.25	3.13	**1.56**	**1.56**	**1.56**
*A. baumannii* 14B00100	1.56	**1.56**	**1.56**	3.13	**1.56**	**1.56**	3.13	6.25	6.25	3.13	**1.56**	**0.78**	**1.56**
*S. aureus* ATCC 25923	1.56	6.25	6.25	12.5	12.5	3.13	12.5	12.5	3.13	6.25	3.13	**1.56**	3.13
*S. aureus* ATCC 33591	3.13	12.5	6.25	50	50	6.25	25	25	50	25	6.25	**3.13**	**3.13**
*S. aureus* ATCC 33592	3.13	6.25	6.25	25	25	12.5	25	25	50	25	6.25	**3.13**	**3.13**
*S. aureus* ATCC 33593	3.13	6.25	6.25	25	25	6.25	25	25	50	25	6.25	**3.13**	**3.13**
*S. aureus* ATCC 6538	1.56	6.25	6.25	3.13	6.25	3.13	6.25	12.5	25	6.25	**1.56**	**1.56**	**1.56**
Bacterial strains	100 mM NaCl												
	W5K/A9W	W5K01	W5K02	W5K03	W5K04	W5K05	W5K06	W5K07	W5K08	W5K09	W5K10	W5K11	W5K12
*E. coli* ATCC 25922	3.13	25	25	50	50	12.5	50	50	12.5	25	12.5	**3.13**	**3.13**
*P. aergionsa* ATCC 27853	1.56	12.5	25	25	25	12.5	25	25	25	12.5	6.25	**1.56**	3.13
*A. baumannii* 14B0091	1.56	3.13	1.56	3.13	3.13	3.13	6.25	6.25	25	12.5	**1.56**	**1.56**	**1.56**
*A. baumannii* 14B0097	1.56	3.13	1.56	3.13	3.13	3.13	6.25	12.5	12.5	12.5	**1.56**	**1.56**	**1.56**
*A. baumannii* 14B00100	1.56	3.13	1.56	3.13	3.13	3.13	6.25	6.25	25	6.25	**1.56**	**0.78**	**1.56**
*S. aureus* ATCC 25923	3.13	12.5	12.5	25	25	6.25	25	25	12.5	12.5	6.25	**3.13**	**3.13**
*S. aureus* ATCC 33591	6.25	12.5	12.5	50	50	12.5	50	50	50	50	**6.25**	**3.13**	**3.13**
*S. aureus* ATCC 33592	3.13	12.5	12.5	50	50	12.5	50	50	50	50	6.25	**3.13**	**3.13**
*S. aureus* ATCC 33593	3.13	12.5	12.5	50	50	12.5	50	50	50	50	6.25	**3.13**	**3.13**
*S. aureus* ATCC 6538	1.56	12.5	12.5	6.25	25	6.25	25	12.5	50	12.5	3.13	3.13	3.13
Bacterial strains	200 mM												
	W5K/A9W	W5K01	W5K02	W5K03	W5K04	W5K05	W5K06	W5K07	W5K08	W5K09	W5K10	W5K11	W5K12
*E. coli* ATCC 25922	3.13	>50	>50	>50	>50	50	>50	>50	50	50	12.5	**3.13**	6.25
*P. aergionsa* ATCC 27853	3.13	>50	>50	25	>50	>50	>50	>50	50	50	12.5	**3.13**	6.25
*A. baumannii* 14B0091	1.56	3.13	3.13	6.25	12.5	6.25	50	50	>50	50	**1.56**	**1.56**	**1.56**
*A. baumannii* 14B0097	1.56	3.13	3.13	6.25	12.5	6.25	50	50	50	50	**1.56**	**1.56**	**1.56**
*A. baumannii* 14B00100	1.56	3.13	3.13	6.25	12.5	6.25	25	25	>50	25	**1.56**	**0.78**	**1.56**
*S. aureus* ATCC 25923	3.13	50	50	>50	>50	50	50	50	50	50	12.5	**3.13**	6.25
*S. aureus* ATCC 33591	6.25	25	50	>50	>50	25	50	50	50	>50	12.5	**6.25**	**6.25**
*S. aureus* ATCC 33592	6.25	25	50	>50	>50	25	>50	>50	>50	50	12.5	**3.13**	**6.25**
*S. aureus* ATCC 33593	6.25	25	50	>50	>50	25	>50	50	>50	>50	12.5	**3.13**	**6.25**
*S. aureus* ATCC 6538	3.13	12.5	25	12.5	50	12.5	50	50	50	12.5	6.25	**3.13**	6.25
Bacterial strains	300 mM												
	W5K/A9W	W5K01	W5K02	W5K03	W5K04	W5K05	W5K06	W5K07	W5K08	W5K09	W5K10	W5K11	W5K12
*E. coli* ATCC 25922	12.5	>50	>50	>50	>50	>50	>50	>50	>50	>50	25	**6.25**	**12.5**
*P. aergionsa* ATCC 27853	6.25	>50	>50	>50	>50	>50	>50	>50	>50	>50	25	**6.25**	12.5
*A. baumannii* 14B0091	1.56	12.5	6.25	25	50	25	>50	>50	>50	>50	3.13	**1.56**	3.13
*A. baumannii* 14B0097	1.56	12.5	6.25	25	50	25	>50	>50	>50	>50	6.25	**1.56**	3.13
*A. baumannii* 14B00100	1.56	6.25	6.25	12.5	25	12.5	>50	50	>50	>50	3.13	**0.78**	**1.56**
*S. aureus* ATCC 25923	6.25	>50	>50	>50	>50	50	>50	>50	>50	>50	25	**6.25**	12.5
*S. aureus* ATCC 33591	12.5	50	50	>50	>50	50	>50	>50	>50	>50	25	**6.25**	**12.5**
*S. aureus* ATCC 33592	12.5	50	>50	>50	>50	50	>50	>50	>50	>50	25	**6.25**	**12.5**
*S. aureus* ATCC 33593	12.5	50	>50	>50	>50	50	>50	>50	>50	>50	25	**6.25**	**12.5**
*S. aureus* ATCC 6538	6.25	25	50	25	>50	25	>50	>50	>50	50	12.5	**6.25**	12.5

Peptides showing MIC values equal to or lower than those of the parent peptide W5K/A9W are highlighted in bold.

**Table 4:** MIC (μg/mL) values for W5KA9W and its derivatives in different concentrations of MgCl_2_.

Bacterial strains	0 mM												
	W5K/A9W	W5K01	W5K02	W5K03	W5K04	W5K05	W5K06	W5K07	W5K08	W5K09	W5K10	W5K11	W5K12
*E. coli* ATCC 25922	1.56	3.13	6.25	6.25	6.25	**1.56**	6.25	12.5	3.13	6.25	3.13	**1.56**	**1.56**
*P. aergionsa* ATCC 27853	1.56	3.13	6.25	6.25	12.5	3.13	6.25	6.25	3.13	3.13	3.13	**1.56**	**1.56**
*A. baumannii* 14B0091	1.56	**1.56**	**1.56**	**1.56**	**1.56**	**1.56**	3.13	3.13	3.13	**1.56**	**1.56**	**1.56**	**1.56**
*A. baumannii* 14B0097	1.56	**1.56**	**1.56**	**1.56**	**1.56**	**1.56**	3.13	3.13	3.13	3.13	**1.56**	**1.56**	**1.56**
*A. baumannii* 14B00100	1.56	**1.56**	**1.56**	**1.56**	**1.56**	**0.78**	3.13	3.13	3.13	3.13	**1.56**	**0.78**	**1.56**
*S. aureus* ATCC 25923	1.56	3.13	6.25	6.25	6.25	1.56	6.25	6.25	**1.56**	3.13	3.13	**1.56**	**1.56**
*S. aureus* ATCC 33591	3.13	6.25	6.25	25	25	6.25	12.5	25	25	25	**3.13**	**3.13**	**3.13**
*S. aureus* ATCC 33592	3.13	6.25	6.25	25	25	6.25	12.5	25	25	25	**3.13**	**3.13**	**3.13**
*S. aureus* ATCC 33593	3.13	6.25	6.25	25	25	6.25	12.5	25	25	25	**3.13**	**3.13**	**3.13**
*S. aureus* ATCC 6538	1.56	3.13	6.25	**1.56**	3.13	3.13	6.25	6.25	12.5	6.25	**1.56**	**1.56**	**1.56**
Bacterial strains	0.5 mM												
	W5K/A9W	W5K01	W5K02	W5K03	W5K04	W5K05	W5K06	W5K07	W5K08	W5K09	W5K10	W5K11	W5K12
*E. coli* ATCC 25922	3.13	6.25	6.25	12.5	12.5	**3.13**	6.25	12.5	6.25	6.25	**3.13**	**3.13**	**3.13**
*P. aergionsa* ATCC 27853	1.56	3.13	6.25	6.25	6.25	3.13	6.25	12.5	3.13	6.25	3.13	**1.56**	**1.56**
*A. baumannii* 14B0091	1.56	**1.56**	**1.56**	**1.56**	**1.56**	**1.56**	3.13	3.13	3.13	3.13	**1.56**	**1.56**	**1.56**
*A. baumannii* 14B0097	1.56	**1.56**	**1.56**	3.13	**1.56**	**1.56**	3.13	3.13	3.13	3.13	**1.56**	**1.56**	**1.56**
*A. baumannii* 14B00100	1.56	**1.56**	**1.56**	3.13	**1.56**	**1.56**	3.13	3.13	3.13	3.13	**1.56**	**1.56**	**1.56**
*S. aureus* ATCC 25923	1.56	3.13	6.25	6.25	6.25	**1.56**	6.25	6.25	3.13	3.13	**1.56**	**1.56**	**1.56**
*S. aureus* ATCC 33591	3.13	6.25	6.25	25	25	6.25	12.5	25	25	25	**3.13**	**3.13**	**3.13**
*S. aureus* ATCC 33592	3.13	6.25	6.25	25	25	12.5	12.5	25	25	25	**3.13**	**3.13**	**3.13**
*S. aureus* ATCC 33593	3.13	6.25	6.25	25	25	12.5	12.5	25	25	25	**3.13**	**3.13**	**3.13**
*S. aureus* ATCC 6538	1.56	1.56	3.13	3.13	3.13	**1.56**	3.13	3.13	3.13	3.13	**1.56**	**1.56**	**1.56**
Bacterial strains	1.5 mM												
	W5K/A9W	W5K01	W5K02	W5K03	W5K04	W5K05	W5K06	W5K07	W5K08	W5K09	W5K10	W5K11	W5K12
*E. coli* ATCC 25922	3.13	6.25	12.5	25	12.5	6.25	6.25	25	6.25	12.5	6.25	**3.13**	**3.13**
*P. aergionsa* ATCC 27853	3.13	6.25	6.25	12.5	12.5	**3.13**	6.25	12.5	6.25	6.25	**3.13**	**1.56**	**1.56**
*A. baumannii* 14B0091	1.56	**1.56**	**1.56**	3.13	3.13	**1.56**	3.13	3.13	3.13	3.13	**1.56**	**1.56**	**1.56**
*A. baumannii* 14B0097	1.56	3.13	3.13	3.13	3.13	**1.56**	3.13	3.13	3.13	3.13	**1.56**	**1.56**	**1.56**
*A. baumannii* 14B00100	1.56	**1.56**	**1.56**	3.13	3.13	**1.56**	3.13	3.13	3.13	3.13	**1.56**	**1.56**	**1.56**
*S. aureus* ATCC 25923	3.13	6.25	6.25	12.5	6.25	**3.13**	6.25	12.5	6.25	6.25	**3.13**	**1.56**	**3.13**
*S. aureus* ATCC 33591	3.13	6.25	6.25	25	25	12.5	12.5	25	50	50	**3.13**	**3.13**	**3.13**
*S. aureus* ATCC 33592	3.13	6.25	6.25	25	25	12.5	25	25	25	25	**3.13**	**3.13**	**3.13**
*S. aureus* ATCC 33593	3.13	6.25	6.25	25	25	12.5	25	25	25	25	**3.13**	**3.13**	**3.13**
*S. aureus* ATCC 6538	1.56	**1.56**	3.13	3.13	6.25	**1.56**	3.13	6.25	6.25	3.13	**1.56**	**1.56**	**1.56**
Bacterial strains	2.5 mM												
	W5K/A9W	W5K01	W5K02	W5K03	W5K04	W5K05	W5K06	W5K07	W5K08	W5K09	W5K10	W5K11	W5K12
*E. coli* ATCC 25922	6.25	12.5	12.5	25	12.5	**6.25**	**6.25**	25	**6.25**	12.5	**6.25**	**3.13**	**3.13**
*P. aergionsa* ATCC 27853	3.13	12.5	6.25	12.5	12.5	6.25	25	25	12.5	12.5	6.25	**3.13**	**3.13**
*A. baumannii* 14B0091	1.56	3.13	**1.56**	3.13	3.13	**1.56**	3.13	3.13	6.25	3.13	3.13	**1.56**	**1.56**
*A. baumannii* 14B0097	1.56	3.13	3.13	3.13	3.13	**1.56**	3.13	3.13	6.25	3.13	3.13	**1.56**	3.13
*A. baumannii* 14B00100	1.56	3.13	3.13	3.13	3.13	**1.56**	3.13	3.13	6.25	3.13	3.13	**1.56**	3.13
*S. aureus* ATCC 25923	3.13	6.25	6.25	12.5	6.25	**3.13**	6.25	12.5	6.25	6.25	**3.13**	**1.56**	**3.13**
*S. aureus* ATCC 33591	3.13	6.25	6.25	50	50	12.5	25	25	50	50	6.25	**3.13**	**3.13**
*S. aureus* ATCC 33592	3.13	12.5	6.25	25	25	12.5	25	25	50	50	**3.13**	**3.13**	**3.13**
*S. aureus* ATCC 33593	3.13	6.25	6.25	50	25	12.5	25	25	50	50	6.25	**3.13**	**3.13**
*S. aureus* ATCC 6538	1.56	3.13	6.25	6.25	6.25	3.13	6.25	6.25	6.25	3.13	**1.56**	**1.56**	**1.56**

Peptides showing MIC values equal to or lower than those of the parent peptide W5K/A9W are highlighted in bold.

**Table 5:** MIC (μg/mL) values for W5KA9W and its derivatives with different levels of pH.

Bacterial strains	pH 7.4												
	W5K/A9W	W5K01	W5K02	W5K03	W5K04	W5K05	W5K06	W5K07	W5K08	W5K09	W5K10	W5K11	W5K12
*E. coli* ATCC 25922	1.56	3.13	6.25	6.25	6.25	**1.56**	6.25	12.5	3.13	6.25	3.13	**1.56**	**1.56**
*P. aergionsa* ATCC 27853	1.56	3.13	6.25	6.25	12.5	3.13	6.25	6.25	3.13	3.13	3.13	**1.56**	**1.56**
*A. baumannii* 14B0091	1.56	**1.56**	**1.56**	**1.56**	**1.56**	**1.56**	3.13	3.13	3.13	1.56	**1.56**	**1.56**	**1.56**
*A. baumannii* 14B0097	1.56	**1.56**	**1.56**	**1.56**	**1.56**	**1.56**	3.13	3.13	3.13	3.13	**1.56**	**1.56**	**1.56**
*A. baumannii* 14B00100	1.56	**1.56**	**1.56**	**1.56**	**1.56**	**0.78**	3.13	3.13	3.13	3.13	**1.56**	**0.78**	**1.56**
*S. aureus* ATCC 25923	1.56	3.13	6.25	6.25	6.25	**1.56**	6.25	6.25	**1.56**	3.13	3.13	**1.56**	**1.56**
*S. aureus* ATCC 33591	3.13	6.25	6.25	25	25	6.25	12.5	25	25	25	**3.13**	**3.13**	**3.13**
*S. aureus* ATCC 33592	3.13	6.25	6.25	25	25	6.25	12.5	25	25	25	**3.13**	**3.13**	**3.13**
*S. aureus* ATCC 33593	3.13	6.25	6.25	25	25	6.25	12.5	25	25	25	**3.13**	**3.13**	**3.13**
*S. aureus* ATCC 6538	1.56	3.13	6.25	**1.56**	3.13	3.13	6.25	6.25	12.5	6.25	**1.56**	**1.56**	**1.56**
Bacterial strains	pH 6.5												
	W5K/A9W	W5K01	W5K02	W5K03	W5K04	W5K05	W5K06	W5K07	W5K08	W5K09	W5K10	W5K11	W5K12
*E. coli* ATCC 25922	1.56	3.13	3.13	3.13	3.13	**1.56**	3.13	6.25	3.13	3.13	**1.56**	**1.56**	**1.56**
*P. aergionsa* ATCC 27853	1.56	**1.56**	3.13	3.13	3.13	**1.56**	3.13	3.13	3.13	3.13	**1.56**	**1.56**	**1.56**
*A. baumannii* 14B0091	1.56	**1.56**	**1.56**	**1.56**	**1.56**	**1.56**	**1.56**	3.13	3.13	**1.56**	**1.56**	**1.56**	**1.56**
*A. baumannii* 14B0097	1.56	**1.56**	3.13	3.13	**1.56**	**1.56**	**1.56**	3.13	3.13	**1.56**	**1.56**	**1.56**	**1.56**
*A. baumannii* 14B00100	1.56	**1.56**	**1.56**	**1.56**	**1.56**	**1.56**	**1.56**	3.13	3.13	**1.56**	**1.56**	**1.56**	**1.56**
*S. aureus* ATCC 25923	1.56	**1.56**	3.13	3.13	3.13	**1.56**	3.13	3.13	**1.56**	**1.56**	3.13	**1.56**	**1.56**
*S. aureus* ATCC 33591	3.13	**3.13**	6.25	12.5	12.5	**3.13**	6.25	12.5	12.5	12.5	**3.13**	**3.13**	**3.13**
*S. aureus* ATCC 33592	3.13	6.25	6.25	12.5	12.5	**3.13**	6.25	12.5	12.5	12.5	**3.13**	**1.56**	**1.56**
*S. aureus* ATCC 33593	3.13	**3.13**	**3.13**	12.5	25	**3.13**	6.25	12.5	12.5	12.5	**3.13**	**1.56**	**1.56**
*S. aureus* ATCC 6538	1.56	**1.56**	3.13	3.13	3.13	**1.56**	6.25	3.13	6.25	3.13	**1.56**	**1.56**	**1.56**
Bacterial strains	pH 5.5												
	W5K/A9W	W5K01	W5K02	W5K03	W5K04	W5K05	W5K06	W5K07	W5K08	W5K09	W5K10	W5K11	W5K12
*E. coli* ATCC 25922	1.56	3.13	3.13	6.25	3.13	**1.56**	6.25	6.25	3.13	3.13	**1.56**	**1.56**	**1.56**
*P. aergionsa* ATCC 27853	1.56	3.13	3.13	6.25	6.25	**1.56**	6.25	6.25	3.13	3.13	3.13	**1.56**	**1.56**
*A. baumannii* 14B0091	1.56	**1.56**	**1.56**	3.13	3.13	**1.56**	3.13	3.13	3.13	3.13	**1.56**	**1.56**	3.13
*A. baumannii* 14B0097	1.56	3.13	3.13	3.13	**1.56**	**1.56**	3.13	3.13	3.13	3.13	**1.56**	**1.56**	3.13
*A. baumannii* 14B00100	1.56	**1.56**	**1.56**	**1.56**	**1.56**	**1.56**	3.13	3.13	3.13	**1.56**	**1.56**	**1.56**	3.13
*S. aureus* ATCC 25923	1.56	3.13	3.13	3.13	6.25	**1.56**	6.25	6.25	3.13	3.13	3.13	**1.56**	3.13
*S. aureus* ATCC 33591	3.13	6.25	6.25	25	12.5	**3.13**	6.25	25	25	12.5	**3.13**	**3.13**	**3.13**
*S. aureus* ATCC 33592	3.13	6.25	6.25	12.5	25	6.25	12.5	25	25	12.5	**3.13**	**3.13**	**3.13**
*S. aureus* ATCC 33593	3.13	**3.13**	6.25	25	25	**3.13**	6.25	12.5	12.5	25	**3.13**	**1.56**	**1.56**
*S. aureus* ATCC 6538	1.56	**1.56**	3.13	3.13	6.25	**1.56**	6.25	6.25	6.25	3.13	**3.13**	**1.56**	**1.56**
Bacterial strains	pH 4.5												
	W5K/A9W	W5K01	W5K02	W5K03	W5K04	W5K05	W5K06	W5K07	W5K08	W5K09	W5K10	W5K11	W5K12
*E. coli* ATCC 25922	1.56	3.13	3.13	6.25	6.25	**1.56**	12.5	12.5	3.13	6.25	3.13	**1.56**	**1.56**
*P. aergionsa* ATCC 27853	1.56	3.13	3.13	12.5	6.25	**1.56**	25	12.5	6.25	6.25	3.13	**1.56**	**1.56**
*A. baumannii* 14B0091	1.56	**1.56**	**1.56**	3.13	3.13	3.13	3.13	6.25	6.25	6.25	3.13	**1.56**	3.13
*A. baumannii* 14B0097	1.56	3.13	3.13	3.13	3.13	3.13	3.13	3.13	6.25	6.25	3.13	**1.56**	3.13
*A. baumannii* 14B00100	1.56	**1.56**	**1.56**	**1.56**	**1.56**	**1.56**	3.13	3.13	3.13	3.13	**1.56**	**1.56**	3.13
*S. aureus* ATCC 25923	3.13	**3.13**	**3.13**	6.25	12.5	**1.56**	6.25	12.5	**3.13**	6.25	**3.13**	**3.13**	**3.13**
*S. aureus* ATCC 33591	3.13	6.25	6.25	25	25	6.25	12.5	25	25	25	6.25	**3.13**	**3.13**
*S. aureus* ATCC 33592	3.13	6.25	6.25	25	25	6.25	12.5	25	25	25	**3.13**	**3.13**	**3.13**
*S. aureus* ATCC 33593	3.13	6.25	6.25	25	25	**3.13**	12.5	25	25	25	6.25	**3.13**	**3.13**
*S. aureus* ATCC 6538	3.13	**3.13**	6.25	6.25	6.25	**3.13**	6.25	6.25	12.5	6.25	6.25	**3.13**	**3.13**

Peptides showing MIC values equal to or lower than those of the parent peptide W5K/A9W are highlighted in bold.

**Table 6:** Therapeutic index(TI) of W5K/A9W and its 12 derivatives in antimicrobial efficiency.

Peptide	GM ^1^			HC10 ^2^	Therapeutic Index (TI) ^3^		
	G− (µg/mL)	G+ (µg/mL)	G−/+ (µg/mL)	µg/mL	G−	G+	G−/+
W5K/A9W	2.1	8.2	4.1	30.0	14.5	3.6	7.3
W5K01	8.2	28.7	15.4	70.9	8.6	2.5	4.6
W5K02	7.2	21.8	12.5	16.7	2.3	0.8	1.3
W5K03	16.5	66.0	33.0	108.7	6.6	1.6	3.3
W5K04	18.9	66.0	35.4	108.7	5.7	1.6	3.1
W5K05	12.5	33.0	20.3	108.7	8.7	3.3	5.4
W5K06	43.5	75.8	57.4	108.7	2.5	1.4	1.9
W5K07	21.8	87.1	43.5	108.7	5.0	1.2	2.5
W5K08	50.0	75.8	61.6	108.7	2.2	1.4	1.8
W5K09	33.0	75.8	50.0	108.7	3.3	1.4	2.2
W5K10	3.1	9.5	5.4	108.7	34.8	11.5	20.0
W5K11	2.1	5.4	3.3	71.0	34.5	13.0	21.2
W5K12	2.4	4.7	3.4	41.7	17.6	8.8	12.4

^1^ GM, geometric mean of the MIC values against Gram-negative bacteria, Gram-positive bacteria, and both. When no detectable antimicrobial activity was observed at 50 µg/mL, a value of 100 µg/mL was used for calculation of the GM value. G−, Gram-negative bacteria. G+, Gram-positive bacteria. G−/+, Both Gram-positive and -negative bacteria. ^2^ HC10 is the minimal inhibitory concentration that induced 10% hemolysis of human red blood cells. When no detectable hemolytic activity was observed at 108.7 µg/mL(200 µM), a value of 108.7 µg/mL was used for calculation of the HC10 value. ^3^ Therapeutic index (TI) is calculated as HC10/GM. Larger values indicate greater cell selectivity.

**Table 7:** Helical content determined based on CD spectra.

Peptide	Helical Content %			
	PBS	TFE	POPC:Cholesterol	POPC:LPS
W5K/A9W	16.7	42.0	20.4	32.8
W5K01	39.5	54.8	62.3	35.8
W5K02	38.9	59.0	30.4	35.5
W5K03	12.8	50.2	5.9	10.0
W5K04	25.7	53.8	10.6	7.5
W5K05	12.2	58.6	4.5	10.8
W5K06	12.2	35.1	8.7	5.9
W5K07	6.2	23.2	8.2	8.6
W5K08	2.6	42.9	12.3	3.9
W5K09	5.3	15.6	5.5	8.4
W5K10	11.4	20.3	8.7	11.1
W5K11	15.3	35.0	8.8	38.0
W5K12	16.7	47.7	5.3	6.8

The helical content was calculated on the website: https://bestsel.elte.hu/index.php, accessed on 13 March 2023.

## Data Availability

No data was used for the research described in the article.

## Supplementary Materials

The following supporting information can be downloaded at: https://www.mdpi.com/article/10.3390/antibiotics14111077/s1, Figure S1: Minimal inhibitory concentration (MIC) values were displayed on a color scale for W5K/A9W and its 12 derivatives under Mueller–Hinton (MH) broth and LYM medium with different concentrations of NaCl. Figure S2: Minimal inhibitory concentration (MIC) values were displayed on a color scale for W5K/A9W and its 12 derivatives under Mueller–Hinton (MH) broth and LYM medium with different concentrations of MgCl_2_. Figure S3: Minimal inhibitory concentration (MIC) values were displayed on a color scale for W5K/A9W and its 12 derivatives under Mueller–Hinton (MH) broth and LYM medium with different levels of pH.

## Abbreviations

The following abbreviations are used in this manuscript:

AMPs	Antimicrobial peptides
ATCC	American Type Culture Collection
BCRC	Bioresource Collection and Research Center
CD	Circular dichroism
CFU	Colony forming unit
DLS	Dynamic light scattering
ELISA	Enzyme-linked immunosorbent assay
HPLC	High performance liquid chromatography
LUV	Large unilamellar vesicles
MH broth	Mueller–Hinton broth
MIC	Minimal inhibitory concentration
MALDI-TOF	Matrix-assisted laser desorption-ionization time-of-flight
MTT	3-(4,5-dimethyl-2-thiazolyl)-2,5-diphenyl-2H-tetrazolium bromide
PBS	Phosphate-buffered saline
POPC	1-palmitoyl-2-oleoyl-sn-glycero-3-phosphocholine
SD	Standard deviation
