# Supplementary material for: Sequence Permutation Generated Lysine and Tryptophan-Rich Antimicrobial Peptides with Enhanced Therapeutic Index
Source: Antibiotics (Basel). 2025 Oct 26;14(11):1077. doi: 10.3390/antibiotics14111077 (PMC12649549; doi:10.3390/antibiotics14111077)
Supplement: Supplementary file 1 [file antibiotics-14-01077-s001.zip › antibiotics-3932628-supplementary.pdf]

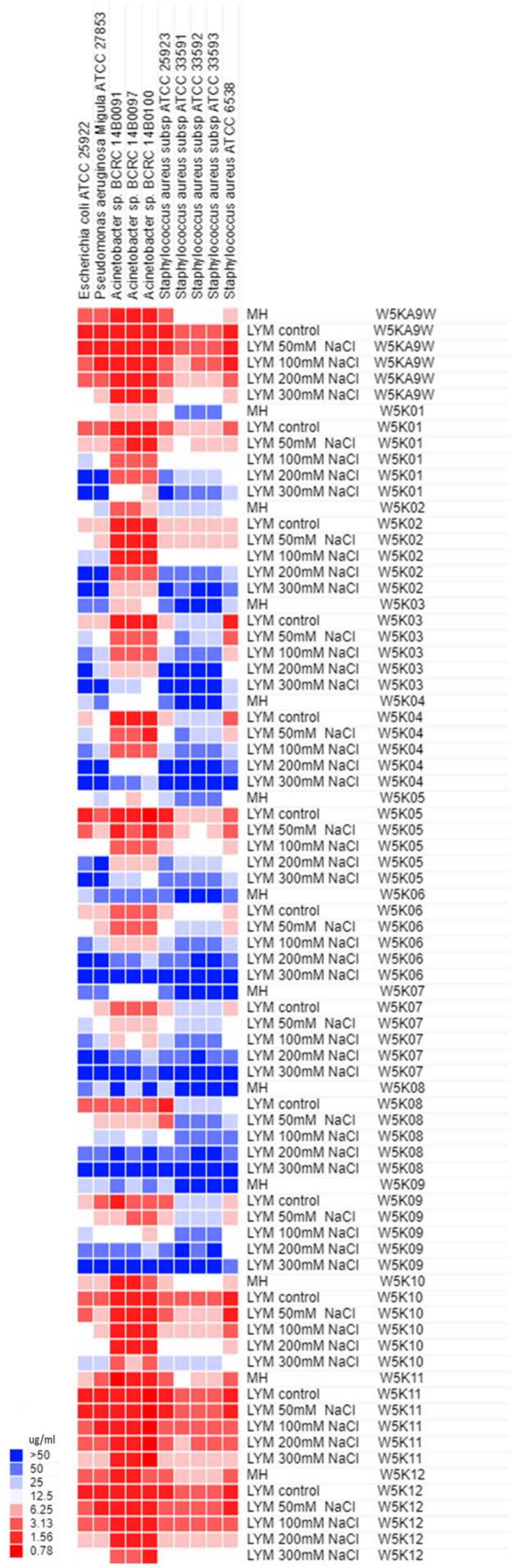

Figure S1. Minimal inhibitory concentration (MIC) values were displayed on a color scale for W5K/A9W and its 12 derivatives under Mueller-Hinton (MH) broth and LYM medium with different concentrations of NaCl.

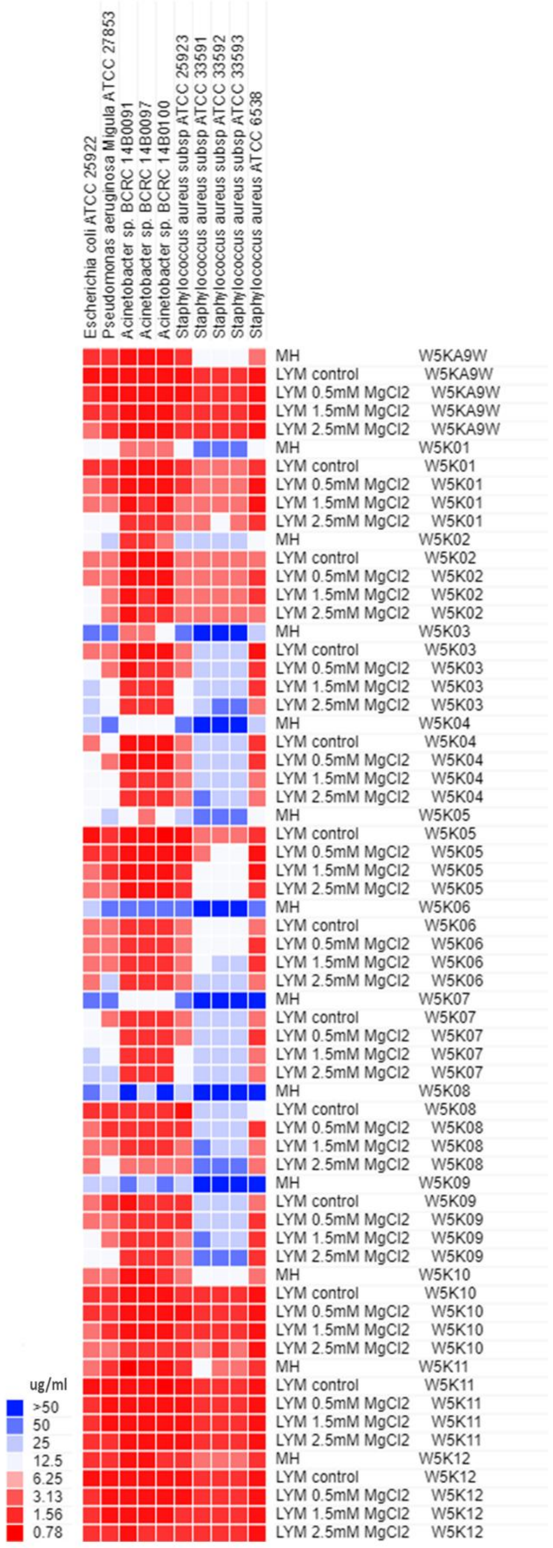

Figure S2. Minimal inhibitory concentration (MIC) values were displayed on a color scale for W5K/A9W and its 12 derivatives under Mueller-Hinton (MH) broth and LYM medium with different concentrations of $MgCl_2$.

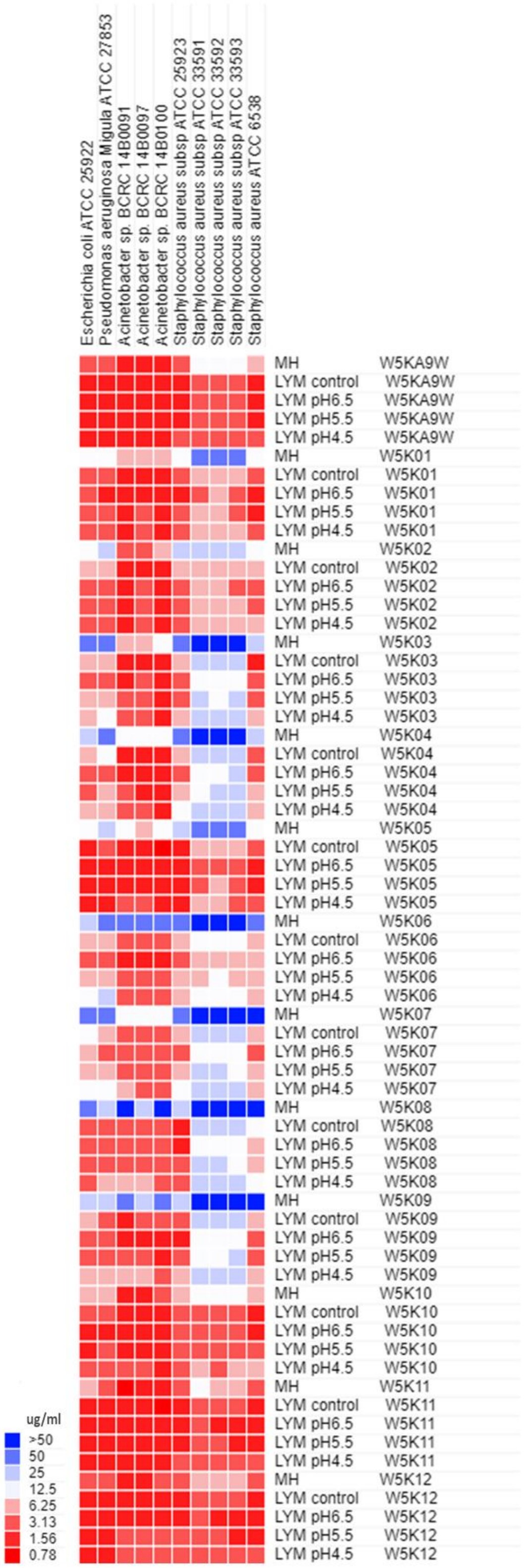

Figure S3. Minimal inhibitory concentration (MIC) values were displayed on a color scale for W5K/A9W and its 12 derivatives under Mueller-Hinton (MH) broth and LYM medium with different levels of pH.
